# Introducing cardiology in numbers

**DOI:** 10.1007/s12471-026-02060-8

**Published:** 2026-07-13

**Authors:** Pim van der Harst

**Affiliations:** Department of Cardiology, University Medical Centre Groningen, Groningen, The Netherlands

With this issue, the Netherlands Heart Journal launches *Cardiology in Numbers*, a recurring series providing epidemiological and clinical figures for the Dutch setting. Such figures are used daily: to inform patients, support grant applications, and outline the rationale of new studies. Yet they are often outdated or drawn from different populations. The series aims to make current, sourced numbers available. It begins with coronary artery disease, reported by Roefs et al. [1]. Further topics will follow in subsequent issues.

This issue continues the NVVC’s series of endorsements of European Society of Cardiology guidelines [2]. Hirsch et al. present working group’s position on the 2024 ESC guidelines for chronic coronary syndromes and application in Dutch practice [3]. The statement addresses the risk-factor-weighted clinical likelihood model, selection of non-invasive imaging, physiological assessment of stenoses during coronary angiography, and management of angina and ischemia with non-obstructive coronary arteries.

This issue includes three original contributions. van de Pol et al. studied more than 1,000 patients with heart failure or atrial fibrillation and found NYHA and EHRA classes correlated with quality of life, indicating these measures do not fully reflect patient experience [4]. van Dinter et al., in a pooled analysis of 8,495 patients, reported postoperative pericardial effusion in 36% but reintervention in only 3%, with large effusions warranting closer follow-up [5]. Voortman et al. evaluated computed tomography in prosthetic valve dysfunction, reporting a 94% negative predictive value for valve-related events at one year and confirmation of abnormal findings in 70% [6].

Together, these articles and the new series reflect the journal’s aim to combine sound data with guidance that supports daily clinical practice.
